# Effects of Monochromatic Light on Mass and Phytocompound Production of *Bacopa monnieri* (L.) Wettst. in the Temporary Immersion System

**DOI:** 10.3390/biology15141116

**Published:** 2026-07-10

**Authors:** Teerawech Promchiangsa, Phatthanan Khiaokhoen, Anupan Kongbangkerd, Boworn Kunakhonnuruk

**Affiliations:** 1 Division of Basic and Medical Sciences, Faculty of Allied Heal Sciences, Pathumthani University, Pathum Thani 12000, Thailand; teerawech.p@ptu.ac.th; 2 Plant Tissue Research Unit, Department of Biology, Faculty of Science, Naresuan University, Phitsanulok 65000, Thailand; phatthanank65@nu.ac.th (P.K.); anupank@nu.ac.th (A.K.)

**Keywords:** Brahmi, bacoside, light quality, HPLC

## Abstract

*Bacopa monnieri* (Brahmi), a valuable medicinal herb, faces challenges in consistent production and quality under natural cultivation. To overcome these limitations, this study investigated the impact of different light qualities (warm-white, red, blue, and green) on the in vitro growth, chlorophyll content, and accumulation of bacosides in *B. monnieri* using a temporary immersion system (TIS). The results revealed that green and blue light significantly enhanced biomass accumulation, with green light promoting the highest number of new shoots (24.2 shoots/clump) and blue light inducing the development of longer shoots with extended internodes (2.6 cm/internode). Interestingly, while green light resulted in the lowest chlorophyll content, it led to the highest accumulation of bacoside A3 (0.39% DW). In contrast, no significant differences were observed in bacopaside II content across all light treatments. However, bacopasaponin C production was significantly enhanced by warm-white and red light. These results demonstrate the potential of manipulating light quality within a TIS to optimize specific growth parameters and bioactive compound production in *B. monnieri*, offering valuable insights for enhancing the quality and yield of this important medicinal plant for industrial applications.

## Simple Summary

This study addresses the challenge of producing high-quality Brahmi (*Bacopa monnieri*), a medicinal plant widely used to enhance memory and reduce anxiety, which often suffers from inconsistent quality when grown naturally. We investigated how different radiation wavelengths of light (warm-white, red, blue, and green) affect the growth and medicinal compound production of this plant when cultured in a liquid culture system. Our findings revealed that green and blue lights are excellent for boosting plant growth, resulting in more shoots and larger plants. Interestingly, while green light reduced the photosynthetic pigment in leaves, it significantly triggered the highest accumulation of bacoside A3. Conversely, warm-white and red lights favored the production of other health-promoting substances. These results demonstrate that carefully selecting light colors in controlled indoor farming can maximize both plant yield and medicinal potency. This approach offers a reliable, chemical-free strategy for the herbal industry to mass-produce high-quality raw materials for health supplements, ultimately benefiting public healthcare.

## 1. Introduction

*Bacopa monnieri* (L.) Wettst., commonly known as Brahmi and a member of the Plantaginaceae family, serves as a versatile herb utilized in both nutrition and traditional medicine. Its phytochemical profile is particularly recognized for its neuroprotective potential, offering therapeutic possibilities for managing memory impairments and neurological conditions. The widespread distribution and ease of propagation of *B. monnieri* across Thailand and other tropical regions position this species as an ideal candidate for large-scale development into nutraceuticals and health-related products [1]. This trend indicates an increasing future demand for high-quality Brahmi raw materials. While *B. monnieri* thrives in humid and aquatic habitats, wild cultivation frequently results in fluctuating biomass yields and non-uniform secondary metabolite profiles, primarily driven by seasonal and environmental instabilities [2]. The presence of heavy metals and hazardous chemicals in natural habitats further complicates the commercial utilization of *B. monnieri* [3]. To overcome these industrial limitations and guarantee pharmacological consistency, there is a critical need for the development of optimized and controlled cultivation methods.

Plant tissue culture is a well-established alternative technique used for propagating both economic and medicinal plants, including Brahmi [4,5]. The reliance on traditional, small-volume containers necessitates a high degree of skilled labor for plantlet transfer, leading to increased labor costs and a high risk of inefficiency. Such spatial and logistical constraints represent a major barrier to the commercial expansion of medicinal plant tissue culture [6,7]. To overcome these constraints, the implementation of plant bioreactor systems offers a streamlined alternative that minimizes manual intervention, reduces operational overheads, and optimizes spatial utilization while significantly boosting mass propagation capacity [8,9]. Specifically, temporary immersion systems (TIS) provide a practical solution for high-throughput cultivation. The intermittent immersion cycles prevent the abnormal growth patterns associated with permanent submersion, fostering healthier and more synchronized plant development [7,8]. Previous studies comparing three culture systems, namely semi-solid culture, conventional liquid culture, and the temporary immersion system (TIS), demonstrated that TIS was the most efficient approach for enhancing growth, development, and secondary metabolite accumulation in *Drosera communis* A.St.-Hil. and *Drosera binata* Labill. [10,11]. In addition to environmental conditions, the spectral composition of light during cultivation serves as a critical regulator of plant developmental plasticity and secondary metabolism [12,13,14]. The effects of light quality are mediated by the specific activation of photoreceptors, including phytochromes, which perceive red and far-red wavelengths, and cryptochromes and phototropins, which perceive blue light. These signaling pathways influence plant growth and metabolism through the regulation of gene expression, transcription factor activity, and the expression of key enzymes involved in metabolic processes [15,16]. These signaling pathways influence plant growth and metabolism through the regulation of gene expression, transcription factor activity, and the expression of key enzymes involved in metabolic processes. Consequently, optimizing light spectra represents a viable approach for tailoring the phytochemical content of in vitro cultures [17,18]. LED light exposure, with different light qualities, significantly enhances the accumulation of triterpenoid saponin glycosides in *B. monnieri* [19,20]. These findings highlight light quality manipulation as a viable strategy for optimizing the biosynthesis of bioactive compounds in medicinal plant cultures. However, no studies have examined the impact of light quality on the growth and development of *B. monnieri* cultivated using TIS. Therefore, this research aims to investigate the influence of light quality on the growth and development of *B. monnieri* in TIS.

## 2. Materials and Methods

### 2.1. Plant Materials

*Bacopa monnieri* plants maintained under aseptic conditions were used as the source of explants. Leaf segments excised from the 3rd to 5th nodes below the shoot apex were cultured on semi-solid Murashige and Skoog (MS) medium [21] supplemented with 0.1 mg/L benzyladenine (BA) and 2 g/L Gel-rite. The culture medium was adjusted to pH 5.8 prior to sterilization by autoclaving at 121 °C for 20 min. Cultures were incubated at 25 °C under warm-white LED illumination with a 12 h photoperiod for 20 days. Adventitious shoot buds subsequently developed along the wounded margins of the leaf explants. The regenerated explants were then utilized as initial plant materials for subsequent experiments.

### 2.2. Effect of Light Quality on Bacopa Monnieri in the Temporary Immersion System (TIS)

The explants were cultivated in a temporary immersion system (TIS) containing plant growth regulator-free MS liquid medium. Immersion was programmed for 10 min every 8 h. A total of 20 explants were cultured in 400 mL of liquid medium within 1000 mL culture vessels [22]. Four LED light treatments (Chihiros WRGB II Slim, Chihiros Aquatic Studio, Cixi, Zhejiang, China), consisting of white light 400–700 nm, blue light 450 nm, green light 530 nm and red light 660 nm, were evaluated, with three replicates assigned to each treatment. Cultures were maintained under a light intensity of 30 μmol m^−2^ s^−1^ using a spectrometer (LI-180 spectrometer, LI-COR Biosciences, Lincoln, NE, USA) and a 12 h photoperiod at 25 °C for four weeks under controlled environmental conditions. Growth performance, new shoot number, and biomass accumulation were subsequently assessed, followed by chlorophyll analysis. Dry biomass was determined after drying the plant clumps in a hot-air oven at 50 °C for 48 h.

### 2.3. Chlorophyll and Carotenoid Measurement

Leaf tissues (0.05 g) were collected from the 2nd to 4th nodes of each plant and homogenized using a mortar and pestle. Subsequently, 1 mL of a chilled extraction solution consisting of 80% acetone and absolute ethanol (1:1, *v*/*v*) was added to the homogenate. The extract was centrifuged at 10,000 rpm for 5 min, and 200 µL of the resulting supernatant was transferred into a 96-well microplate. Chlorophyll content was determined using a microplate reader (Synergy H1, Agilent Technologies, Winooski, VT, USA) by measuring absorbance at OD_441_, OD_645_, and OD_663_ nm. The recorded absorbance values were subsequently used to calculate chlorophyll concentrations according to the equations described by [23]. Chlorophyll a = [(12.25 × OD_663_) − (2.55 × OD_645_)] × [1/(100 × W)]; chlorophyll b = [(20.30 × OD_645_) − (4.91 × OD_663_)] × [1/(100 × W)]; total chlorophyll = [(7.34 × OD_663_) + (17.76 × OD_645_)] × [1/(100 × W)]; carotenoid = [(4.46 × OD_441_) − Chl a + Chl b] × [1/(100 × W)], where W refers to the weight of the fresh leaves (g).

### 2.4. Bacopa Monnieri Extraction for HPLC Analysis

Dried shoot samples (0.1 g) were transferred into 2 mL screw-cap tubes and preheated in a hot-air oven at 45 °C for 60 min. The samples were subsequently pulverized using a BeadBug™ Microtube Homogenizer (Benchmark Scientific, Sayreville, NJ, USA) operating at 300 rpm for 90 s. Following homogenization, 1 mL of methanol was added, and the mixture was vortex-mixed for 1 min before sonication at 40 °C for 15 min using an Ultrasonic Bath Sonicator (Ravi Scientific Industries, Delhi, India). The extract was centrifuged at 10,000 rpm for 3 min, and the resulting supernatant was collected into a fresh microcentrifuge tube. The extraction procedure was performed twice to ensure complete recovery of target compounds. The combined methanolic extracts (2 mL) were concentrated in a hot-air oven at 45 °C for 24 h. The final volume was adjusted to 1 mL, after which the extract was passed through a 0.45 µm nylon syringe filter and transferred into a 1.5 mL vial. Prior to analysis, all extracts were stored at −80 °C. Bacoside quantification was carried out using high-performance liquid chromatography coupled with diode-array detection (HPLC-DAD) (Agilent 1100 Series, Agilent Technologies, Frankfurt am Main, Germany). Chromatographic separation was achieved on a Purospher^®^ STAR RP-18 column (250 × 4.6 mm, 5 µm particle size, Merck KGaA, Darmstadt, Germany). The mobile phase consisted of acetonitrile and 0.2% aqueous phosphoric acid (65:35, *v*/*v*), delivered at a flow rate of 1.0 mL min^−1^ following a 60 min column equilibration period. Bacoside compounds were identified and quantified by comparing their retention times with those of a bacoside reference standard, with detection performed at 205 nm [22].

### 2.5. Statistical Analysis

The experiment was designed using a Completely Randomized Design (CRD). The data were analyzed for variance using ANOVA, and the differences between means were compared using Duncan’s New Multiple Range Test (DMRT) at a 95% confidence level (*p* ≤ 0.05) by IBM SPSS statistics version 25.

## 3. Results

### 3.1. The Growth and Development of Bacopa Monnieri Under Different Light Qualities

**Figure 1:**
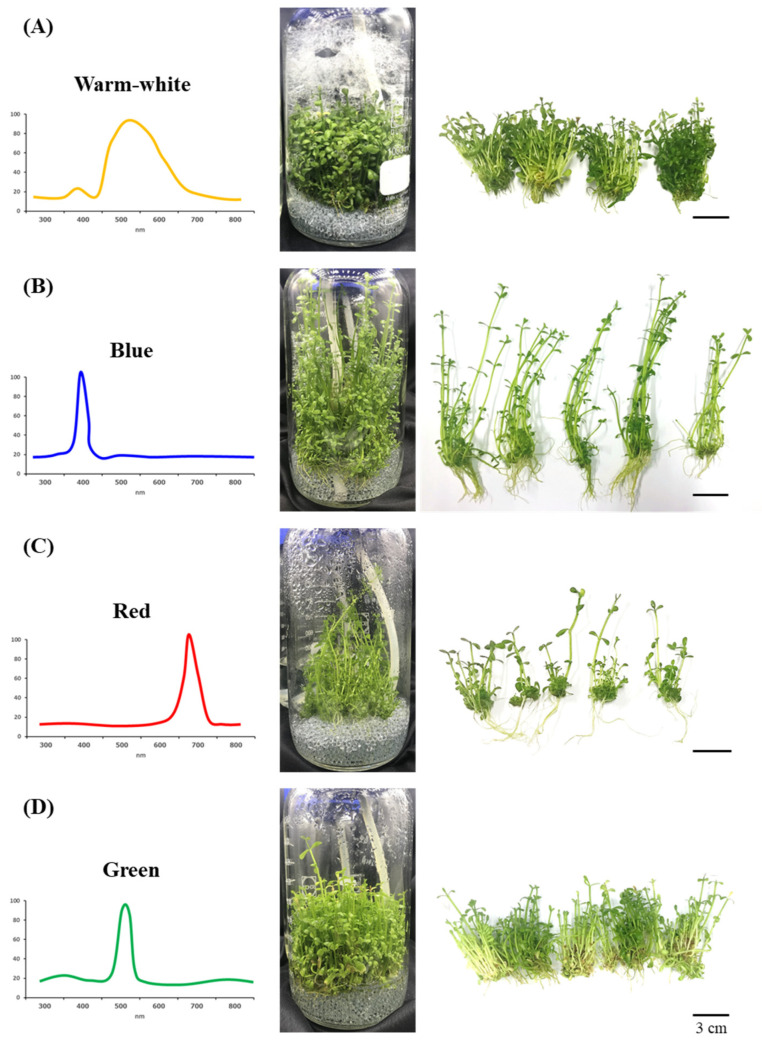
The growth and development of *Bacopa monnieri* explants in a temporary immersion system (TIS) after four weeks of culture under different light qualities: (**A**) Warm-white light (400–700 nm); (**B**) Blue light (450 nm); (**C**) Red light (660 nm); and (**D**) Green light (530 nm).

**Figure 2:**
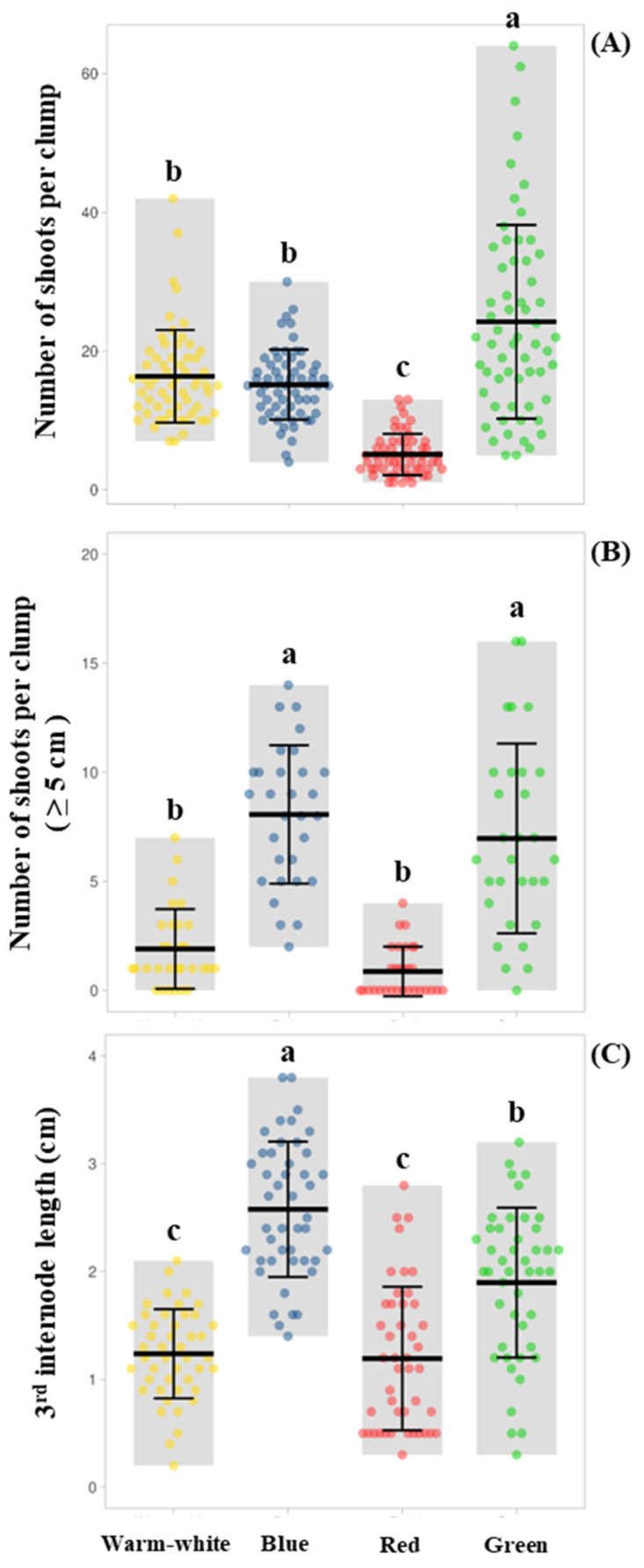
Box plots illustrating (**A**) total shoots per clump, (**B**) number of shoots ≥ 5 cm, and (**C**) mean 3rd internode length. Data represent means from 60 plantlets. Bars with different letters indicate significant differences according to Duncan’s Multiple Range Test (DMRT) at *p* ≤ 0.05.

*B. monnieri* explants were cultured under different light qualities (warm-white, green, blue, and red) for four weeks using a temporary immersion system (TIS). All explants survived and grew regardless of the light quality, and no abnormalities, such as waterlogging, were observed (Figure 1). Explants exposed to green and blue light showed the highest fresh weight increase per clump (1.77 g/clump and 1.62 g/clump, respectively). However, when comparing fresh weights of the stems and leaves, green and blue light resulted in the highest fresh weight (0.91 g FW/clump and 0.81 g FW/clump) and dry weight (0.07 g DW/clump and 0.06 g DW/clump). In contrast, explants exposed to red light exhibited the lowest growth and biomass accumulation (0.13 g FW/clump and 0.01 g DW/clump), which was significantly different from the other light treatments. Regarding the total dry weight per culture container, explants exposed to warm-white, blue and green light had the highest biomass (1.02 g, 1.16 g and 1.46 g/container, respectively) (Table 1). Observing the formation of new shoots, the green light treatment resulted in the highest number of new shoots (24.2 shoots/clump), significantly (Figure 2A). However, explants exposed to blue light produced new shoots with at least 5 cm height (9 shoots/clump), which also resulted in the longest internodes in new shoots (2.6 cm/internode) (Figure 2B,C).

### 3.2. Chlorophyll and Carotenoid Analysis

**Figure 3:**
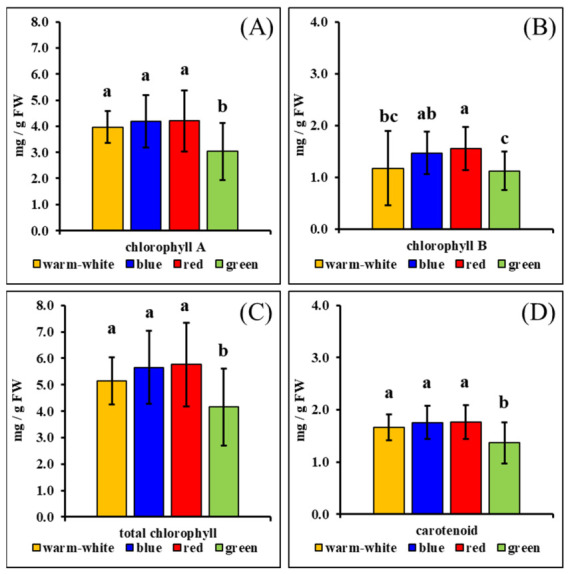
Photosynthetic pigments in *Bacopa monnieri* cultured under different light qualities for four weeks: (**A**) chlorophyll A; (**B**) chlorophyll B; (**C**) total chlorophyll and (**D**) carotenoid. Different letters indicate significant differences, analyzed by Duncan’s Multiple Range Test (DMRT) at *p* ≤ 0.05.

Following four weeks of culture in a Temporary Immersion System (TIS) using hormone-free MS liquid medium, photosynthetic pigment variations were observed in *B. monnieri* under different light qualities. The chlorophyll analysis of Brahmi (*Bacopa monnieri*) using a microplate reader were found that *B. monnieri* cultured under warm-white, blue and red light had the highest chlorophyll A content (3.97, 4.19 and 4.21 mg/g FW, respectively), Similarly, chlorophyll B content showed under red (1.56 mg/g FW), followed by blue (1.47 mg/g FW) and warm-white light (1.18 mg/g FW). Consequently, these three treatments yielded the highest total chlorophyll contents, standing at 5.76, 5.66, and 5.14 mg/g FW for red, blue, and warm-white lights, respectively. A parallel trend was observed in the carotenoid analysis, where red (1.77 mg/g FW), blue (1.76 mg/g FW), and warm-white lights (1.66 mg/g FW) consistently sustained elevated concentrations. In contrast, *B. monnieri* cultured under green light showed the lowest in all photosynthetic pigments (Figure 3).

### 3.3. The Quantities of Bacoside in Bacopa Monnieri Under Different Light Qualities

**Figure 4:**
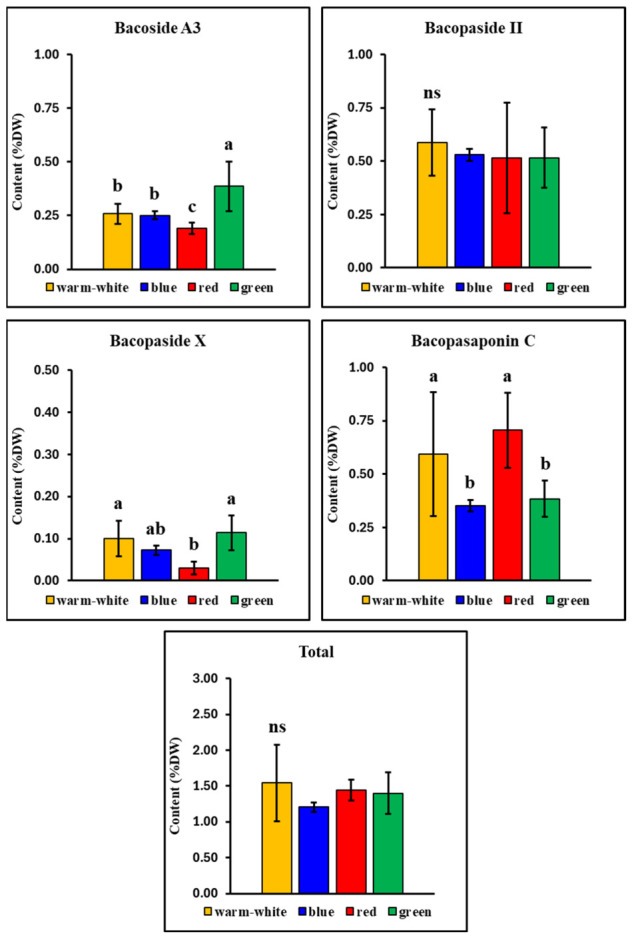
The bacoside content of *Bacopa monnieri* cultured in a temporary immersion system (TIS) under different light qualities for four weeks. Different letters indicate significant differences, analyzed by Duncan’s Multiple Range Test (DMRT) at *p* ≤ 0.05.

Bacoside content in *B. monnieri* was quantified after a four-week culture period. High-performance liquid chromatography (HPLC) was employed for bacoside analysis, with sample chromatograms compared against a mixed bacoside standard. *B. monnieri* cultured under green light exhibited the highest bacoside A3 content (0.39% DW). A relatively high bacoside A3 content (0.26% DW and 0.25% DW), also statistically similar to the control, was observed under warm-white and blue light. Conversely, red light significantly reduced bacoside A3 content (0.19% DW) (Figure 4). Regarding bacopaside II, the highest content (0.59% DW) was measured in the warm-white light treatment, though this was not statistically different from other light conditions (Figure 4). Bacopaside X content was highest in both the warm-white light (0.10% DW) and green light (0.11% DW) treatments. Red light yielded the lowest bacopaside X content (0.03% DW) (Figure 4). Interestingly, bacopasaponin C content was significantly higher in *B. monnieri* cultured under warm-white light (0.59% DW) and red light (0.71% DW) compared to green light (0.38% DW) and blue light (0.35% DW). However, no significant differences were observed among treatments for bacopaside II and total bacoside content (Figure 4).

## 4. Discussion

Plants exhibit species-specific responses to in vitro light conditions [24,25,26]. To enhance both plant growth and the production of secondary metabolites, it is important to optimize light conditions, which encompass photoperiod, intensity, and light quality [17,18,27]. Cultivating Brahmi (*Bacopa monnieri*) using a temporary immersion system (TIS) under different light qualities (warm-white, blue, red, and green) revealed that blue and green light resulted in new shoots in height and produced the longest internodes among warm-white and red light conditions. This finding confirms that blue and green light trigger the shade-avoidance response, a process which plants elongate to escape shading by other plants [20,28,29]. In addition to green and blue light, there exist photoreceptors capable of absorbing light across two spectral regions (320–500 nm), which play a crucial role in regulating photomorphogenesis. These include cryptochromes and phototropins, which are involved in controlling plant growth processes, mediating phototropic responses, and enhancing photosynthetic efficiency [30]. Conversely, some researchers have indicated that the impact of red light on plant height varies according to species [17,31]. Under red light, *B. monnieri* produced fewer new shoots, resulting in lower fresh and dry weights. This outcome is attributed to the absence of essential wavelengths that are critical for maintaining cytokinin levels, a hormone vital for cell division. Consequently, the deficiency in red light leads to fewer shoots, short shoot length and diminished biomass [13,20]. Although red light induced fewer new shoots, resulting in lower fresh and dry weights, it yielded photosynthetic pigment content similar to that of blue and warm-white light, both of which resulted in higher biomass and shoot numbers.

In contrast, green light resulted in the lowest pigment levels but encouraged the greatest number of new shoots and biomass relative to other light conditions, which is adequate for plant development. When considering bacoside production, the results revealed that green light significantly enhanced bacoside A3 accumulation compared to all other light treatments. This effect was particularly pronounced relative to the red light treatment, which yielded less than 50% of the bacoside A3 levels observed under green light. Interestingly, the warm-white and blue light treatments exhibited an intermediate response; both spectra stimulated significantly higher bacoside A3 accumulation than red light, yet remained significantly lower than the levels achieved under green light. Many reports suggest that green light can be beneficial in plant cultivation, stimulating growth development and promoting increased secondary metabolite production [20,32]. This may be attributed to the significant role of green light in carbon fixation during photosynthesis, as well as its importance in promoting biomass accumulation within the deeper layers of the leaf. Green light can penetrate lower cellular layers due to its relatively low absorption by chlorophyll referred to as the green window and its enhanced scattering within leaf tissues [33]. Recent research suggests that green light can cause *Arabidopsis thaliana* to exhibit shade avoidance phenotypes [34]. Kaiser et al. [35] found that higher proportions of green light in natural sunlight significantly increased tomato stem length and specific leaf area. Cao et al. [36] reported that green light stimulates ginger height by upregulating the expression of genes associated with gibberellin biosynthesis. These findings indicate that the gibberellin pathway plays a role in mediating the effect of green light on plant height. This hypothesis is supported by similar observations in *Arabidopsis thaliana* under different light conditions, where green light-induced hypocotyl elongation was found to be independent of known photoreceptors [37].

Our study, employing a temporary immersion system for in vitro *B. monnieri* cultures, has conclusively demonstrated that both blue and green light play a crucial role in regulating both biomass and bacoside production. These findings suggest that blue and green light were particularly effective for promoting specific *B. monnieri* characteristics, such as internode elongation, while different light spectra influence the production of distinct secondary metabolites. Therefore, a strategic combination of light conditions, specifically incorporating blue and green light, offers a promising approach to optimizing the growth and development of *B. monnieri* for enhanced production of desired compounds.

## 5. Conclusions

The effects of different light qualities on *Bacopa monnieri* growth and phytochemical production were investigated over four weeks in a temporary immersion system (TIS). Our results demonstrate that blue and green light significantly enhance several growth parameters, including clump fresh weight, aerial part fresh and dry weight and the number of new shoots. Furthermore, blue light specifically promoted internode elongation. While green light resulted in lower photosynthetic pigment production compared to other light treatments. Regarding phytochemical production, light quality exercised a profound effect on specific bacoside accumulation. Monochromatic green light significantly enhanced the biosynthesis of bacoside A3, achieving the highest yield and outperforming both the warm-white and blue light, while red light resulted in the lowest accumulation. Conversely, warm-white and red lights favored the accumulation of bacopasaponin C. These findings highlight the strategic potential of utilizing specific LED wavelengths, particularly green light, in TIS bioreactors to simultaneously optimize vegetative yield and maximize the accumulation of high-value memory-enhancing compounds for industrial applications.

## Figures and Tables

**Table 1:** Biomass of *Bacopa monnieri* cultured in a temporary immersion system (TIS) under different light qualities for four weeks.

Light Qualities	Clump FW (g/Clump)	Shoot Weight (g/Clump)		Total DW (g/Container)
		FW	DW	
warm-white	1.30 ± 0.04 b ^1^	0.64 ± 0.02 b	0.05 ± 0.00 b	1.02 ± 0.17 a
blue	1.62 ± 0.06 a	0.81 ± 0.04 a	0.06 ± 0.00 a	1.16 ± 0.18 a
red	0.37 ± 0.04 c	0.13 ± 0.02 c	0.01 ± 0.00 c	0.20 ± 0.06 b
green	1.77 ± 0.15 a	0.91 ± 0.08 a	0.07 ± 0.01 a	1.46 ± 0.62 a

^1^ Different letters within the same column indicate significant differences, analyzed by Duncan’s Multiple Range Test (DMRT) at *p* ≤ 0.05. Values are means ± SE of 3 replications except for total dry weight as mean ± SD of 3 replications.

## Data Availability

The data related to the findings of this research are available upon request from the corresponding author.

## Abbreviations

The following abbreviations are used in this manuscript:

TIS	Temporary immersion system
HPLC	High-Performance Liquid Chromatography
MS	Murashige and Skoog media
BA	Benzyladenine
FW	Fresh weight
DW	Dry weight
